# Anti-Infective Activity of *Momordica charantia* Extract with Molecular Docking of Its Triterpenoid Glycosides

**DOI:** 10.3390/antibiotics13060544

**Published:** 2024-06-11

**Authors:** Mohammed Aldholmi, Rizwan Ahmad, Mohammad Habeeb Shaikh, Ayad Mohammed Salem, Maher Alqurashi, Mansour Alturki

**Affiliations:** 1 Department of Natural Products, College of Clinical Pharmacy, Imam Abdulrahman Bin Faisal University, Dammam 31441, Saudi Arabia; 2 Department of Physiology, College of Medicine, Imam Abdulrahman Bin Faisal University, Dammam 34224, Saudi Arabia; 3 Department of Microbiology, College of Medicine, Imam Abdulrahman Bin Faisal University, Dammam 31441, Saudi Arabia; 4 Department of Pharmaceutical Chemistry, College of Clinical Pharmacy, Imam Abdulrahman Bin Faisal University, Dammam 31441, Saudi Arabia

**Keywords:** karavilosides, antiviral, antibacterial, antifungal, bitter melon

## Abstract

*Momordica charantia*, commonly known as bitter melon, is a fruiting plant that has been used for several diseases including infectious diseases. In this study, we report the antibacterial, antifungal, and antiviral activity of different bitter melon fruit parts originating from India and Saudi Arabia. The in vitro experiments are supported by the molecular docking of karavilosides to verify their role in the bioactivity. The antimicrobial assays revealed activity against *Candida albicans*, *Escherichia coli*, and *Staphylococcus aureus*. The extracts exhibited the potent inhibition of HIV-I reverse transcriptase, with an IC50 of 0.125 mg/mL observed for the pith extract originating from Saudi Arabia and the standard drug doxorubicin. The molecular docking of karavilosides exhibited a significant affinity to reverse transcriptase comparable to Rilpivirine and higher than that of doxorubicin. These outcomes encourage the precious bioactive components of the seed and pith of the Saudi bitter melon fruits to be further studied for isolation and structure elucidation.

## 1. Introduction

Infectious diseases caused by pathogens, including viruses, bacteria, fungi, and parasites, are still a substantial risk to public health, despite the availability of numerous classes of anti-infective agents. Some reports estimated that by 2050, ten million people will die every year from antimicrobial resistance, unless a universal response is projected to tackle this problem [1,2]. Many of the approved anti-infective agents are becoming less effective as the pathogens develop resistance mechanisms to avoid the effect of the utilized drugs, while other medications have crucial issues related to their pharmacokinetic and pharmacodynamic properties, as well as leading to unpleasant side effects [3,4]. Therefore, exploring novel anti-infective agents with unique mechanisms of action and fewer side effects is a research propriety supported by the governments to combat infectious diseases. Natural compounds from plants, microorganisms, and marine organisms represent an essential source of anti-infective agents. It has been estimated that 66% of all small-molecule anti-infective drugs approved by the FDA between January 1981 and September 2019 are either natural products or based on natural products pharmacophore [5].

Traditional medicinal plants have been used for over fifty centuries as a source of antimicrobial, anticancer, analgesic, cardioprotective, antihypertensive, antidiabetic, and other medications [6]. Plants produce bioactive compounds known as secondary metabolites with a chemical structural diversity. Examples of the major classes of bioactive secondary metabolites include alkaloids, phenolics, and terpenoids. Tamiflu (oseltamivir) is an antiviral phenolic-based medication for influenza A and B produced from the shikimic acid obtained from star anise [7]. The traditional Chinese *Artemisia annua*, commonly known as sweet wormwood, is the source of the antimalarial terpenoid artemisinin, which has saved the lives of millions of people around the world [8]. Terpenoids have been reported to demonstrate potent antiviral activity and reduce viral infectivity [9,10]. For instance, artemisinin and its derivatives have been reported to show antiviral activity against various viruses, including the human cytomegalovirus (HCMV), Hepatitis B virus (HBV), and Hepatitis C virus (HCV) [11]. A synergetic effect was noticed when these terpenoids were combined with the antiviral lamivudine, which might be an effective strategy to minimize the serious adverse effects of the latter.

*Momordica charantia*, known as bitter melon or bitter gourd, is a fruiting vine belonging to the family Cucubitaceae, probably native to tropical and subtropical Africa and Asia [12]. The plant is wildly cultivated in India, China, and Southeast Asia. It is used as a vegetable crop, as well as a medicine against several diseases, including diabetes, cancer, dementia, hyperlipemia, hypertension, digestive problems, and infectious diseases [12]. The ethanol extract of *M. charantia* leaves was found to have an antibacterial activity against *Escherichia coli*, *Staphylococcus aureus*, *Bacillus subtilis*, *Pseudomonas aeruginosa*, *Salmonella typhi*, and *Klebsiella pneumonia* [13,14]. The antifungal activity of the leaves extract was also reported to work against *Candida albicans* [15]. The leaves extract has also shown activity against *Aeromonas hydrophila*, the most common pathogenic bacteria of freshwater fish [16]. The ground seeds of bitter melon were shown to present an antibacterial activity against *Pasteurella multocida* and *Staphylococcus aureus* [17]. The antimicrobial activity was evaluated using disk diffusion; hence, the minimum inhibitory concentration and minimum bactericidal concentration were not reported. Several proteins, e.g., MAP 30, alpha and beta-momorcharin, and lectin, have been isolated from the Chinese bitter melon seeds and are reported to have an antiviral activity against coxsackievirus B3, human immunodeficiency virus (HIV), herpes, Epstein–Barr, and polio viruses [18,19,20]. The antiviral activity of MAP30, alpha-momorcharin, and beta-momorcharin was attributed to the inhibition of HIV-1 integrase [18]. The bitter melon lectin was reported to show its antiviral bioactivity via the inhibition of the reverse transcriptase enzyme [21]. Another protein known as MRK29 was isolated from the Thai bitter melon seed and reported to act through the same mechanism [22]. Nevertheless, there are limited studies on the antiviral, antibacterial, and antifungal activity of bitter melon fruits and the role of karavilosides in this bioactivity, particularly for reverse transcriptase inhibition. Moreover, most previous studies have been limited to the Chinese and Thai bitter melon, while the bitter melon commonly used in other Asian countries originates from India. There are also local varieties of bitter melon cultivated in most countries and consumed, along with the imported ones.

In this context, we evaluated the antiviral, antibacterial, and antifungal activity of different parts of bitter melon fruits to determine the specific part responsible for bioactivity. Our study included one local origin (Saudi Arabia) and one global origin (India) of BM fruits to compare the bioactivity of the fruits produced by plants grown under different climatic and environmental conditions. Finally, a detailed molecular docking study of the major phytochemicals of the BM fruits (karavilosides; KVs) was performed to evaluate the role of KVs in the antiviral activity via the inhibition of reverse transcriptase enzyme.

## 2. Results

### 2.1. Extract Yield and KV Quantity

The highest extract yield was observed for K1 (5.41%) and I1 (2.78%), followed by I3 (1.18%), K3 (0.56%), K2 (0.18%), and I2 (0.09%). The extracts with the highest total amount of KVs (KV-VIII, -X, and -XI)/BM fruit part were I3 (1217.81 µg/L), K3 (970.67 µg/L), and I2 (734.17 µg/L). On an individual basis, KV-VIII (592.1 µg/L) and KV-XI (405.32 µg/L) were the highest in I3, while the highest amount of KV-X was found in K3 (269.55 µg/L). The details regarding extract yields and KV quantities for all the samples are provided in Table S1.

### 2.2. Antiviral Activity

The non-radioactive HIV-RT colorimetric ELISA kit revealed an antiviral activity for the BM extracts, with a mean inhibition (±SD) of 76.35 (±3.46%) against HIV-I reverse transcriptase. The inhibition (%) for HIV-RT was observed in the range of 72.40–81.60, where K1 exhibited the greatest inhibition of 81.6%, followed by K3 (79.2%). The standard drug doxorubicin showed an inhibition of 89.6% in the study. The inhibition data for all the samples (I1-I3 and K1-K3) against HIV-I RT are shown in Table S1. The active extract of K1 was further evaluated for the IC_50_ calculation, where a similar IC_50_ of 0.125 mg/mL was observed for K3 and the standard drug doxorubicin. The IC_50_ data, along with the negative and positive controls for HIV-RT, are shown in Table S2. The descending order for the antiviral activity of the extracts may be summarized as K1 (81.6%) > K3 (79.2%) > I2 (76%) > I3 (75.2%) > K2 (73.7%) > I1 (72.4%).

### 2.3. Antimicrobial Activity (MIC and MBC)

The antimicrobial assays revealed minimum inhibitory concentrations (MICs) of the extracts in the range of 0.08–1.0 mg/mL against *Candida albicans* (CA), 0.3–3.0 mg/mL against *Escherichia coli* (EC), and 3.0–3.0 mg/mL against *Staphylococcus aureus* (SA). The minimum bactericidal concentrations (MBCs) ranged between 0.08 and 1.0 mg/mL against *C. albicans*, 1.0 and 3.0 mg/mL against *E. coli*, and 3.0 and >3 mg/mL for *S. aureus*. The detailed antimicrobial activity for all the samples (I1-I3 and K1-K3) against bacteria and yeast is shown in Table S1.

On an individual basis, the lowest MIC (0.08 mg/mL) was observed for the I1 and I3 extracts against *C. albicans*, followed by 0.3 mg/mL for I2 and K3 against *C. albicans*. The lowest MIC against *E. coli* (0.3 mg/mL) was observed for I1, I3, and K3, whereas all the I and K samples presented a similar MIC of 3 mg/mL against *S. aureus*. For MBC against *C. albicans*, the lowest concentration of 0.08 mg/mL was observed for I3, followed by 0.3 mg/mL for I1 and I2. The lowest MBC against *E. coli* was observed to be 1 mg/mL for I1, I3, and K3. All the samples exhibited MBCs of 3 or >3 mg/mL against *S. aureus*. The details regarding the MIC and MBC for all the samples against *C. albicans*, *E. coli*, and *S. aureus* are provided in Table S1. The ascending order for the MICs and MBCs is summarized as *C. albicans*_MIC (I1 and I3) = *C. albicans*_MBC (I3) > *C. albicans*_MIC (I2 and K3) = *C. albicans*_MBC (I1, I2, and K3) > *E. coli*_MIC (I1, I3, and K3) > (MIC/MBC for the remaining samples against *E. coli* and *S. aureus*).

### 2.4. Molecular Docking Studies

**Figure 1:**
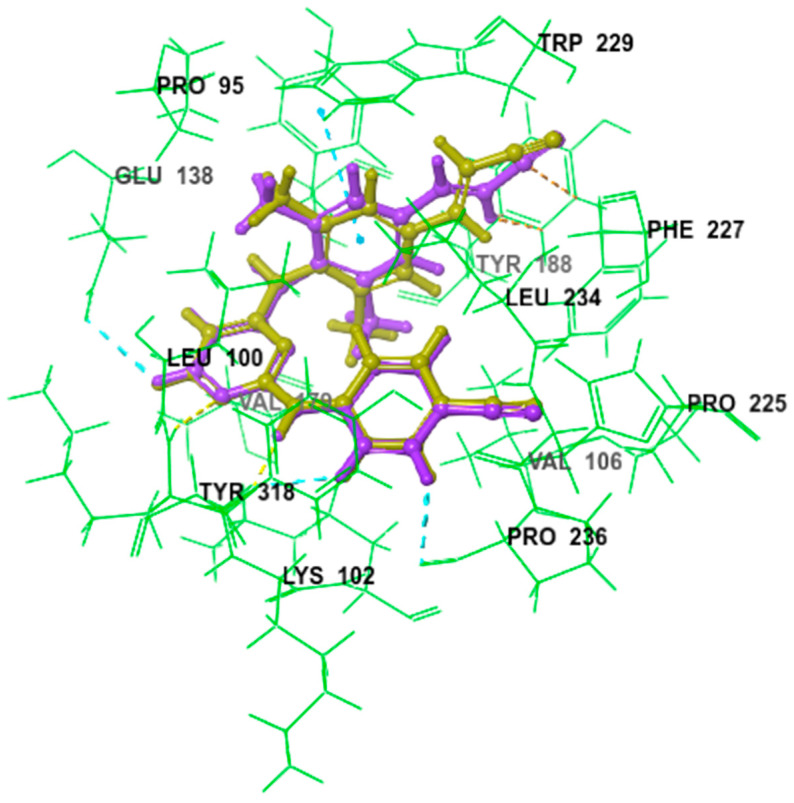
Comparative binding positions of cognate inhibitor (light green) and redocked inhibitor (purple) bound to RT active site, RMSD of 0.3571.

The ligands were docked using the extra precision mode (XP) without any constraints and a 0.80 van der Waals (vdw) radius scaling factor and a 0.15 partial charge cut-off were used. The GlideScore, implemented in Glide, was used to estimate the binding affinity and to rank the ligands, whereas the XP-Pose rank was used to select the best-docked pose for each ligand. The compounds were then thoroughly analyzed based on the binding scores along with a comprehensive examination of all the binding interactions. The results revealed good binding modes of superimposition, with an RMSD of 0.3571, which initially reflects the accuracy of pose prediction for the Glide (Figure 1).

**Figure 2:**
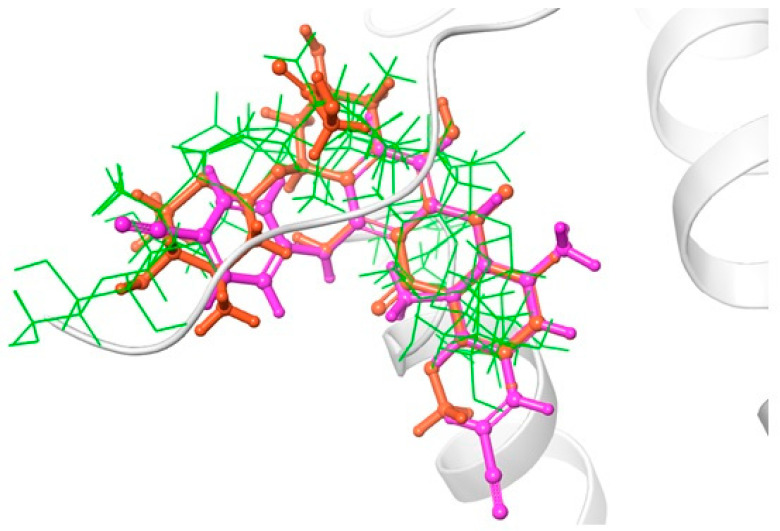
Comparative binding positions of Rilpivirine (magenta); doxorubicin (orange); and karavilosides VIII, X, and XI (green), bound to RT active site.

The karavilosides ligands were then docked in the RT active site using the extra precision mode on Glide [9]. The results showed that all karavilosides ligands adapted a relatively similar docking pattern in the active site that is close to the reference drug Rilpivirine, and doxorubicin poses, which suggests the potential affinity of these molecules to the RT active site (Figure 2). The calculated RMSD for the best binding poses in reference to Rilpivirine and doxorubicin ranged from 0.0.5 to 0.2 for karavilosides VIII, X, and XI. The relative binding affinities (Glide score) of the docked compounds on the enzyme were analyzed, and the results showed that karaviloside XI has the highest glide score for the inhibition of HIV-1 reverse transcriptase (Table 1).

### 2.5. Statistical Models

#### 2.5.1. Correlation Analysis

Pearson’s bivariate model was applied to establish the significant correlations for the samples’ origin vs. dependent variables (DVs) in the dataset. For sample origin vs. DV, a positive correlation (*p* = 0.05) was seen for the pairs of I2 and DV (0.670; 0.02), I3 and DV (0.638; 0.03), and K3 and DV (0.620; 0.04). The correlation within the samples showed a positive correlation (*p* = 0.01) for I1 and K1 (0.997; 0.00), whereas I2, I3, K2, and K3 revealed a lack of any correlation with I1 and K1. The details for the pairwise correlation for the sample origin and DV is given in Table S3.

#### 2.5.2. Principal Component Analysis (PCA)

**Figure 3:**
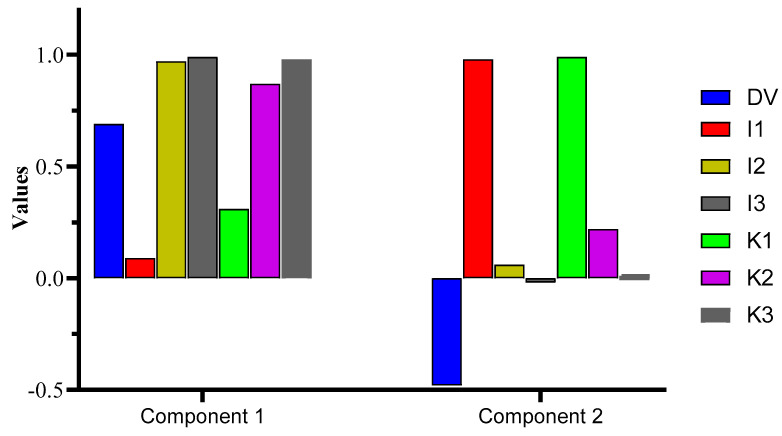
PCA representation of the components loaded with DVs in component 1 and 2.

The principal component analysis (PCA) exhibited a cumulative variance of 90.52%, where PC1 computed the major variance of 58.82%, followed by PC2 (31.70%). Interestingly, PC1 was loaded with DV, I2, I3, K2, and K3, whereas I1 and K1 were loaded in PC2. The outcome confirms Pearson’s test data, i.e., a greater correlation was observed for DV vs. the I2, I3, K2, and K3 samples, which is confirmed herein by the greater variance in the data shown by the mentioned samples vs. DV. The test was significant at *p* = 0.05, with a high X2 value of 141.33. The % variance for the components with detailed data regarding the KMO and Bartlett’s test is shown in Figure 3 and Table S4.

#### 2.5.3. Paired Samples *t*-Test

The dataset was evaluated for pair differences using the sample origins vs. individual biological activities for the six extracts. The data revealed only three pairs with significant pair differences—antiviral activity t(−60.32) at *p* = 0.00 (CI = −75.95–69.75), MIC for *C. albicans* t(4.40) at *p* = 0.01 (CI = 1.27–4.82), and MBC for *C. albicans* t(4.21) at *p* = 0.01 (CI = 1.17–4.84). The remaining data were inconsiderable, as shown in Table S5.

## 3. Discussion

**Figure 4:**
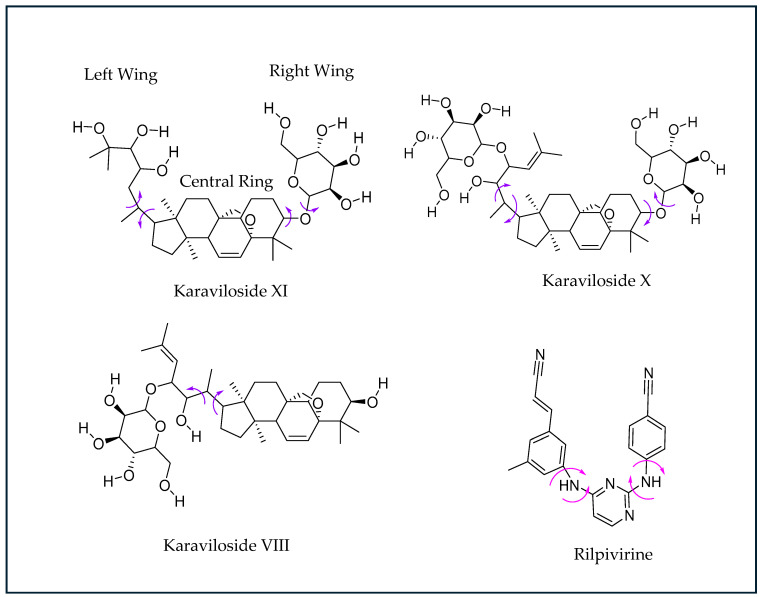
Chemical structures of NNRTI Rilpivirine (RPV). The torsion angles defining the rotatable bonds are labeled using purple-colored arrows in Karaviloside VIII, X, and XI. The equivalent torsion angle RPV is labeled using magenta-colored arrows. The structures of karavilosides, apart from karaviloside VIII, can be divided into three functional regions—a steroidal central ring, a hexose sugar/ethylene glycol left wing, and a hexose sugar right wing.

This study investigated the antiviral and antimicrobial potential of three different parts (skin, seeds, and pith) of the BM fruits from two distinct origins of India (I) and the Kingdom of Saudi Arabia (K). Following the green extraction and LCMSMS quantitative analysis for the presence of the three different cucurbitane triterpenoid glycosides (karavilosides; KVs), i.e., KV-VIII, -X, and -XI in these BM fruit parts, molecular docking was executed to determine the affinity and binding properties for the KVs. The ligands for the KV compounds exhibited a significant affinity to the reverse transcriptase (RT) protein. To broaden the findings, the RT inhibition profile for the KVs was studied through docking in the active site of the named target. The binding pattern, target interactions, and binding affinities for the KVs were investigated and compared to the reference drugs Rilpivirine and doxorubicin. For the validation of the docking phenomenon, the process was repeated via the redocking of the co-crystalline ligand in their respective target (HIV-1 reverse transcriptase) while utilizing the alike protocols, as applied for the KV ligands. Subsequently, rigid-body superposition was performed for the predicted lowest energy conformation of the target with its corresponding crystalline ligand using the structure superposition tool on Maestro. The classical root mean square deviation (RMSD) for the predicted binding poses from the co-crystalline pose was calculated at an RMSD < 2Å cut-off value [23]. The detailed analysis for the calculated binding affinities of the KVs exhibited a comparable inhibition to Rilpivirine and higher than that of doxorubicin. A horseshoe conformation was seen for the KVs during RT binding (Figure 4), which is similar to the NNRTIs in the DAPY family [24,25].

**Figure 5:**
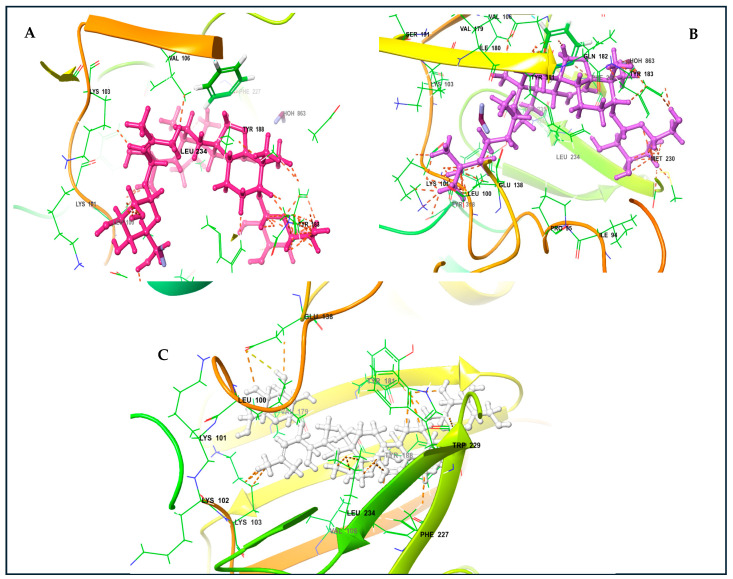
Karavilosides bound to RT active site. (**A**) Karaviloside X. (**B**) Karaviloside XI. (**C**) Karaviloside VIII.

The KVs exhibited an impressive structural complementarity to the non-nucleoside inhibitor-binding pocket (NNIBP). Moreover, KV-X and -XI (left-wing structures of these compounds) interact with GLU138, LYS103, VAL106, VAL179, and LEU100 through van der Waals (VDW) interactions. The presence of the steroidal ring (center) enables the establishment of Pi and non-polar interactions with Phe227 and LEU234. Likewise, the right wing for these compounds interacts with TYR181, TYR188, and MET230 (Figure 5A,B). For KV-VIII, less interaction was observed with the non-nucleoside inhibitor-binding pocket (Figure 5C). The variation in affinity and bindings may be due to the absence of the third functional region compared to KV-X and -XI. A superior binding affinity for the protein binding based on the Pi and non-polar interactions with GLU138, LYS103, VAL106, VAL179, and LEU100 have been reported for KV-XI [26].

The findings from the molecular docking suggested valuable insights into the probable role of KVs in combating the ongoing challenges in HIV treatment. The six extracts were evaluated against HIV-I RT, where K1 and K3 revealed the highest inhibition of the HIV-I RT. The extract with the highest inhibition (K1) was compared to the standard drug doxorubicin to determine the IC_50_ value. Both tested samples were seen with relatable IC_50_ values, suggesting the potential inhibitory role for K1 against HIV-I RT. With regard to the BM fruit, these extracts represent the seeds (K1) and pith (K3) parts of the fruit. The correlation with an in-depth analysis of the extract yield and phytochemical profile established a paramount link between the KV amount in these samples. Although the K1 sample had the lowest amount of KVs, a high extract yield among the samples was reported for K1. This favors the exploration of the K1 sample to investigate the presence of other phytochemical classes and to establish the biological activity reported herein. Though antimicrobial and anti-HIV activities have been reported for bitter melon metabolites, several triterpenoid glycosides are present in the fruit and might contribute to the overall bioactivity [27]. Therefore, a detailed study of the mentioned phytochemicals is required to link them to the anti-HIV activity of the BM fruit.

On the contrary, the K3 sample was quantified with the highest amount of KV-X on an individual basis, as well as the highest amounts of the total KVs (KV-VIII, -X, and -XI) in an individual fruit part, following I3. Previous studies reported the antiviral role of the BM against herpes, Epstein–Barr, and HIV [28,29], where the juice from BM fruit was effective in suppressing HIV via an enhanced T-cell count and a stabilization of the CD4/CD8 cells in the HIV-positive patients. This anti-HIV role was demonstrated by the α- and β-momorcharin proteins present in the BM seed. In addition, the presence of anti-retroviral protein (MAP30) was studied for the probable anti-HIV, anti-HBV, and anti-herpes activities of the BM [28,30]. Yet again, there is a lack of research to establish the expected role of KV-VII, -X, and -XI in HIV inhibition. The isolation with appropriate in-depth molecular-level studies may be helpful in uncovering the novel role of these KVs. The outcome of the antiviral study herein supports the traditional use of BM in treating viral infections [27,31].

In order to explore the antimicrobial potential, the extracts were screened against the fungal strain of *C. albicans*, as well as the bacterial strains of *E. coli* and *S. aureus*. For the antimicrobial demarcation, the results for MIC and MBC manifested a substantial activity against *C. albicans*, followed by a significant MIC against *E. coli*. However, the MIC and MBC values for *S. aureus* exhibited insignificant results for the extracts. In contrast to the antiviral activity, the lowest MIC value against *C. albicans* was noted for I1 and I3, followed by K3, whereas the lowest MBC value against *C. albicans* was recorded for I3 only. Likewise, the lowest MIC values against *E. coli* were seen for I1, I3, and K3 among the six extracts. The comprehensive analysis of these extracts reveals a high extract yield for I1, following K1. The I3 extract contains the highest amount of both, the total KVs (KV-III, -X, -XI), along with the amount of KV-VIII and -XI in an individual sample among the six extracts. The K3 extract has been mentioned previously to contain the highest amount of KV-X on an individual basis. Herein, the seeds and pith parts of the BM fruit also presented antifungal and antibacterial activities, which underscore the significance of these parts of the BM fruits in anti-infective activities.

The samples with significant antimicrobial activity observed were I1 and I3. For I3, though an enormous amount of KVs was observed during quantitative analysis, no studies have reported the KVs’ correlation with antimicrobial activities, which entails the study for an individual antimicrobial model where the KVs’ potential is explored against these species with appropriate mechanisms. For I1, there was a high extract yield with the least detected amount of KVs, suggesting the presence of other phytochemicals in the extract, imparting an antifungal activity to the extract. A study reported the presence of flavonoids in the BM leaves with significant antimicrobial activity [32]; however, the phenolics and flavonoids content in the fruit parts remain unexplored. Likewise, the presence of trans-nerolidol in the seeds’ essential oil has been reported for the antimicrobial potential of the BM fruit [33]. The results for the antimicrobial activity in this study are in line with the reported literature. However, a detailed characterization of the I1 and I3 extracts may foster the presence of novel chemical entities in the BM fruit parts. The outcomes in the shape of KVs’ correlation with the biological activity are a call for the important prospect of the KVs in having antiviral and antifungal activities to be able to establish the essential role of these KVs in the emerging and resistant diseases of microbial and HIV infections.

In terms of geographical origin, a significant antiviral activity was witnessed for the KSA sample, whereas the Indian sample revealed a substantial activity against *C. albicans* and *E. coli*. Both the fruits and their parts were noted with sizeable differences in the extract yield and KV amounts. The disparity may be due to the change in climate, air, soil, salinity, altitude, and various geographical factors, as previously reported [34,35]. A detailed investigation of the effect of these factors may be helpful in categorizing the optimum conditions for the growth and marketing of the BM fruit with the maximum extract yield, KV amount, and bioactivity. Nevertheless, the exciting commonality observed in the study was that the seeds and pith parts from the fruits of both origins exhibited an intriguing antiviral and antimicrobial activity. The authors are of the opinion that the KVs were the critical contributors to these activities, irrespective of the nature of the KVs, i.e., KV-VIII, -X, or -XI present in these samples.

To depict the biological activities with the KVs profile of the BM fruit, statistical tests were computed for the dependent variables (DVs) to highlight the significant correlations and differences among the different origin extracts. The bivariate Pearson analysis displayed a significant pairwise correlation for I3 and K3 vs. DVs at *p* = 0.05, whereas I1 and K1 exhibited a positive correlation at *p* = 0.01. I1 and K1 showed the highest extract yields, whereas I3 and K3 had the highest KV amounts, in addition to their antiviral and antimicrobial activities. The PCA further confirmed the loading with more % variability for DVs with I3 and K3 in PC1. The extracts for I1 and K1 were loaded in PC2 with less % variability. This suggests the seed parts with higher extract yield and the pith parts enriched with the KVs are responsible for the respective activities observed in the study. Though the seed part revealed the lowest KVs among the fruit parts, an in-depth exploration is certainly recommended to investigate the chemical compounds present in these extracts, which play a pivotal role in the tested activities. The exploration may be a source of promising novel drug discovery from natural products in order to combat the challenges faced during HIV treatment and to alleviate the burden of microbial infections.

Moreover, the sample origin was tested against the individual biological activity using the two-tailed paired *t*-test. The pairs with significant *p*-values observed were sample origin vs. antiviral and antifungal activity against *C. albicans*. The statistical models finalized the seeds and pith parts from the BM fruit with significant antiviral activity against HIV and antifungal activity against *C. albicans*, suggested due to the presence of KVs.

## 4. Methods

### 4.1. Sample Collection and Preparation

Green and fresh BM fruits (Saudi and Indian origins) were purchased from the local farm house and markets in the Eastern province of Saudi Arabia and were prepared as previously reported in our LCMS/MS quantitative study [36]. Briefly, the fruits were sliced to remove the seeds and pith parts, while the skin part of the fruit was carefully abrased to separate it from the pith part using a sharp blade. The fruit parts were then placed on an aluminum sheet and dried in an oven at 60 °C.

### 4.2. Extraction and Analysis of KVs (Karavilosides)

The green ultrasound-assisted extraction with liquid chromatography–tandem mass spectrometry (LCMSMS) quantification for the three KVs (KV-VIII, -X, and -XI) using different parts (skin, seeds, and pith) of the bitter melon fruit (BM) from two different origins (India; I, and Saudi Arabia; K) have been reported in our previous study [36]. Concisely, one gram of each fruit part was subjected to ultrasonic extraction with a 1:1 mixture of acetone and ethanol. The extracted samples were dried and used for final % extract yield calculation and LCMSMS analysis. The details regarding extract yields (%) and KVs amount (µg/L) for the six extracts of the BM fruit—I1 (Indian seeds extract), I2 (Indian skin extract), I3 (Indian pith extract), K1 (Saudi Arabia seeds extract), K2 (Saudi Arabia skin extract), and K3 (Saudi Arabia pith extract)—are given in detail in Table S1.

### 4.3. Antiviral Activity

The antiviral activity was investigated against the specific drug target known as reverse transcriptase enzyme, a key enzyme in the retroviral life cycle, particularly for human immunodeficiency virus (HIV). The antiviral activity of *Momordica charantia* fruit part extracts was evaluated using a non-radioactive HIV-RT colorimetric ELISA kit (Roche Diagnostics, Mannheim, Germany). The protocol outlined in the kit was followed, using 2 ng of the enzyme in a well and incubating the reaction for 2 h at 37 °C [37]. The extracts were diluted with assay buffer and were tested at 2 mg/mL. The assay was carried out in duplicate, and the absorbance measurement of samples was carried out utilizing a microplate reader at 405 nm wavelength (ELISA; BioTek, Winooski, VT, USA). The negative control (water instead of sample) and positive control (Doxorubicin—Zydus Celexa, Ahmedabad, India) [38,39] were tested under the same conditions. The emerging color intensity is directly related to the reverse transcriptase action. The percentage of reverse transcriptase inhibition was calculated by comparing the absorbance of the samples to the negative control using the equation below [40]:

(1) $$\mathrm{HIV}-1\;\mathrm{RT}\;\mathrm{inhibition}\;\left(\%\right)=\left\{1-\left(\frac{\mathrm{sample}\;\mathrm{absorbance}}{\mathrm{negative}\;\mathrm{control}\;\mathrm{absorbance}}\right)\right\}\times \;100$$

The extract showing the highest inhibition was investigated further, along with doxorubicin at concentrations ranging from zero to 2 mg/mL, to obtain a dose–response curve. The IC_50_ value for the extract and positive control were calculated by plotting the inhibition % against different concentrations using GraphPad Prism software (version 8.0) [40].

### 4.4. Antimicrobial Activity

#### 4.4.1. Microbial Strains and Media

The bacterial strains consisted of *Staphylococcus aureus* (ATCC-25923), *Escherichia coli* (ATCC-25922), and *Candida albicans* (ATCC-14053). The culture media used for the agar-well diffusion method were blood (SPLM; cat-1009), MacConkey (Criterion; cat-C16131), and Sabouraud dextrose agar (SAB; criterion cat C6811), whereas Muller–Hinton broth (MHB, Oxoid, CM0405) was used for the broth dilution method to determine both MIC (minimum inhibitory concentration) and MBC (minimum bactericidal concentration).

#### 4.4.2. Standard Inoculum

The selected colonies from the blood-, MacConkey-, and SAB-grown microorganisms (37 °C; 24 h) were inoculated in Muller–Hinton broth to form a homogenous microbial suspension and were standardized up to 0.5 McFarland turbidity (DensiCHEK plus, bioMérieux, Taguig, Philippines).

#### 4.4.3. Determination of MIC and MBC

The six selected extracts (I1, I2, I3, K1, K2, and K3) were serially diluted with Muller–Hinton broth in a 96-well microtiter plate and were mixed to provide eight different concentrations for the assays in the range of 0.0005–3 mg/mL. The 0.5 McFarland standard (50 µL) for the bacterial strains in MHB was poured into each well of the six selected extracts, as well as into the control. The plates were incubated overnight at 37 °C, and the MIC and MBC were calculated as per the guidelines of the Clinical and Laboratory Standards Institute [41]. All the antimicrobial results were analyzed using Microsoft Excel 2021 and GraphPad Prism 8.0 software.

### 4.5. Molecular Docking Studies

#### 4.5.1. Molecular Modeling Optimization

The computational simulations were performed using the Maestro 13.6; Schrödinger 2023-2, LLC, New York, NY, USA. The crystal structure of the studied enzyme, i.e., wild-type HIV-1 reverse transcriptase (RT), was retrieved from the Research Collaboratory for Structural Bioinformatics (RCSB) Protein Data Bank (PDB), with a corresponding PDB ID of 4G1Q and a resolution of 1.51 Å [42]. The crystal structure was prepared using the Schrödinger Maestro’s Protein preparation wizard tool at a pH of 7.4, with the correction of ionization states, the addition of polar hydrogens, and the removal of the non-essential water molecules. The entire structure was minimized and optimized with the OPLS3 force field to optimize protein energies and remove any steric hindrance. The default RMSD value of 0.30 Å was used for non-hydrogen atoms. In order to prepare the ligand, Maestro Ligprep was used, where the SDF format structures were downloaded and converted to Maestro’s 3D format. Epik, with several treatments, was utilized to determine the optimal chirality and ionization states at a physiological pH of 7.4 ± 2.0. Finally, the geometries were optimized using the OPLS3 force field. These conformations were used as the initial input structures for docking [43].

#### 4.5.2. Binding Pocket Determination and Validation of Molecular Docking

The binding pocket was identified by the workspace co-crystallized inhibitor, non-nucleoside reverse transcriptase inhibitor (NNRTI), and Rilpivirine using Schrödinger’s Maestro. A docking grid was created using the Glide software; Schrödinger 2023-2, LLC, New York, NY, USA, which was identified via a ligand-binding pocket from the crystal structure that was co-crystallized with the inhibitor. To ensure accuracy, the co-crystallized inhibitor was redocked using the alike protocol order. The docking poses and interactions were verified using maestro structure superimposition and root mean square deviation calculations (RMSD) of the alignment [23,44]. The receptor grids were generated using a 1.0 van der Waals (vdw) radius scaling factor, a 0.25 partial charge cut-off, and they were centered on the bound inhibitor. The binding site was enclosed within the grid box using default parameters and without constraints. Finally, the docking process was repeated and verified using three screening settings.

### 4.6. Statistical Analysis

SPSS V 22.0 and GraphPad Prism 8 were used to analyze the data and create the graphs. The results are presented as the mean ± standard deviation (SD) from three experiments. All data were analyzed with a *p*-value of <0.05 considered as significant in order to find the statistical significance between treated groups and controls.

## 5. Conclusions

The BM fruit samples from two different origins were investigated for their antiviral and antimicrobial role based on the KVs amount quantified using LCMSMS. The extracts exhibited >70% inhibition of HIV-RT, with K1 and K3 showing the highest inhibition (≈80%) among the samples. For the antimicrobial activity, the lowest MIC and MBC were observed for I3, I1, and K3 against *C. albicans* and *E. coli*. The correlation studies for the KVs and biological activities in these extracts established a prospective impact for the KVs in K3 and I3. For K1 and I1, the presence of other chemical compounds seems to impart biological activities to the extracts. The bioactive compounds might be other karaviloside derivatives or entirely different chemical entities. These outcomes unfold the crucial role of the seeds and pith parts of the BM fruits to be further studied for the isolation and identification of the novel molecules from these fruit parts. This may result in the discovery of novel medications to alleviate the disease burden of HIV and resistant microbial infections and to improve the patients’ quality of life in the community.

## Figures and Tables

**Table 1:** Docking energies of investigated karavilosides (KV-VIII, -X, and -XI) and co-crystallized reference controls at the binding site of HIV-1 reverse transcriptase (PDB entry: 4G1Q), throughout the preliminary docking analysis. A higher negative value indicates higher binding interactions within the binding pocket.

Compounds	Glide Score Docking Energy (Kcal/mol)	Free Binding Energy (Kcal/mol)	H-Bond Interactions	Pi- Interactions	Van Der Waals Interactions
KV-VIII	−9.213	−60.74	LEU100 and GLU138	PHE227, TYR181, TYR188 and LYS103	GLU138, PHE227, LYS103, PHE227 and LEU234
KV-X	−11.829	−76.91	TYR181, TYR188 and MET230	TYR181 and PHE227	GLU138, PHE227, LYS103, VAL106 VAL179, LEU100 and LEU234
KV-XI	−12.732	−93.23	TYR181, TYR188, TRP229 and LYS101	TYR181 and PHE227	GLU138, PHE227, LYS103, VAL106, VAL179, LEU100 and LEU234
Rilpivirine	−13.350	−116.07	LYS101 and GLU138	TYR181	PRO236 and LYS101
Doxorubicin	−8.352	−40.34	LEU100, LYS103, GLU138, GLY99 and ILE180	PHE227	TYR188, LEU234 and VAL179

## Data Availability

All data generated or analyzed during this study are included in this published article.

## Supplementary Materials

The following supporting information can be downloaded at: https://www.mdpi.com/article/10.3390/antibiotics13060544/s1, Table S1. Antiviral and antibacterial results for BM fruit parts with extract yields and KV amounts (* I1: Indian seeds; I2: Indian skin; I3: Indian pith; K1: KSA seeds; K2: KSA skin; K3: KSA pith). Table S2. IC50 determination for the active extract based on the antiviral inhibition results. Table S3. Bivariate correlation for the extracts vs. DVs at *p* = 0.05. * Correlation is significant at the 0.05 level (2-tailed). ** Correlation is significant at the 0.01 level (2-tailed). Table S4. Principial component analysis for the DVs with component loadings and KMO and Bartlett’s test at *p* = 0.05. Table S5. Paired differences for the sample origin vs. individual activity at *p* = 0.05.

## Abbreviations

HCMV	human cytomegalovirus
HBV	Hepatitis B virus
HCV	Hepatitis C virus
BM	bitter melon
HIV	human immunodeficiency virus
LCMSMS	liquid chromatography–tandem mass spectrometry
KVs	karavilosides
I	India
K	Kingdom of Saudi Arabia
RT	reverse transcriptase
ELISA	enzyme-linked immunosorbent assay
SAB	Sabouraud dextrose agar
MHB	Muller–Hinton broth
MIC	minimum inhibitory concentration
MBC	minimum bactericidal concentration
CA	*Candida albicans*
EC	*Escherichia coli*
SA	*Staphylococcus aureus*
RCSB	Research Collaboratory for Structural Bioinformatics
PDB	Protein Data Bank
NNRTI	non-nucleoside reverse transcriptase inhibitor
RMSD	root mean square deviation
vdw	van der Waals
SD	standard deviation
PCA	principal component analysis
GLU	glutamic acid
LYS	lysine
VAL	valine
LEU	leucine
Phe	phenylalanine
TYR	tyrosine
MET	methionine

## References

[1] De Kraker M.E.A., Stewardson A.J., Harbarth S. (2016). Will 10 Million People Die a Year due to Antimicrobial Resistance by 2050?. PLoS Med..

[2] O’Neill J. (2014). Antimicrobial Resistance: Tackling a Crisis for the Health and Wealth of Nations.

[3] Okeke I.N., Laxminarayan R., Bhutta Z.A., Duse A.G., Jenkins P., O’Brien T.F., Pablos-Mendez A., Klugman K.P. (2005). Antimicrobial resistance in developing countries. Part I: Recent trends and current status. Lancet Infect. Dis..

[4] Aldholmi M., Marchand P., Ourliac-Garnier I., Le Pape P., Ganesan A. (2019). A Decade of Antifungal Leads from Natural Products: 2010–2019. Pharmaceuticals.

[5] Newman D.J., Cragg G.M. (2020). Natural Products as Sources of New Drugs over the Nearly Four Decades from 01/1981 to 09/2019. J. Nat. Prod..

[6] Anand U., Jacobo-Herrera N., Altemimi A., Lakhssassi N. (2019). A Comprehensive Review on Medicinal Plants as Antimicrobial Therapeutics: Potential Avenues of Biocompatible Drug Discovery. Metabolites.

[7] Patra J.K., Das G., Bose S., Banerjee S., Vishnuprasad C.N., del Pilar Rodriguez-Torres M., Shin H. (2020). Star anise (*Illicium verum*): Chemical compounds, antiviral properties, and clinical relevance. Phyther. Res..

[8] Su X.-Z., Miller L.H. (2015). The discovery of artemisinin and the Nobel Prize in Physiology or Medicine. Sci. China Life Sci..

[9] Astani A., Reichling J., Schnitzler P. (2010). Comparative study on the antiviral activity of selected monoterpenes derived from essential oils. Phyther. Res..

[10] Guo Y., Ma A., Wang X., Yang C., Chen X., Li G., Qiu F. (2022). Research progress on the antiviral activities of natural products and their derivatives: Structure–activity relationships. Front. Chem..

[11] Wohlfarth C., Efferth T. (2009). Natural products as promising drug candidates for the treatment of hepatitis B and C. Acta Pharmacol. Sin..

[12] Gayathry K.S., John J.A. (2022). A comprehensive review on bitter gourd (*Momordica charantia* L.) as a gold mine of functional bioactive components for therapeutic foods. Food Prod. Process. Nutr..

[13] Ingle A., Kapgatte R. (2018). Phytochemical screening and antimicrobial activity of *Momordica charantia* Linn. Int. J. Pharmacol. Res..

[14] Leelaprakash G., Rose J.C., Gowtham B.M., Javvaji P.K., Prasad S.A. (2011). In vitro antimicrobial and antioxidant activity of *Momordica charantia* leaves. Pharmacophore.

[15] Jagessar R.C., Mohamed A., Gomes G. (2008). An evaluation of the antibacterial and antifungal activity of leaf extracts of *Momordica charantia* against *Candida albicans*, *Staphylococcus aureus* and *Escherichia coli*. Nat. Sci..

[16] Masithoh D.A., Kusdarwati R., Handijatno D. (2019). Antibacterial activity of bitter gourd (*Momordica charantia* L.) leaf extract against Aeromonas hydrophila. IOP Conf. Ser. Earth Environ. Sci..

[17] Mahmood A., Raja G.K., Mahmood T., Gulfraz M., Khanum A. (2012). Isolation and characterization of antimicrobial activity conferring component (s) from seeds of bitter gourd (*Momordica charantia*). J. Med. Plants Res..

[18] Grover J., Yadav S. (2004). Pharmacological actions and potential uses of *Momordica charantia*: A review. J. Ethnopharmacol..

[19] NG T.B., WONG C.M., LI W.W., YEUNG H.W. (1986). Isolation and characterization of a galactose binding lectin with insulinomimetic activities. Int. J. Pept. Protein Res..

[20] Yao X., Li J., Deng N., Wang S., Meng Y., Shen F. (2011). Immunoaffinity purification of α-momorcharin from bitter melon seeds (*Momordica charantia*). J. Sep. Sci..

[21] Wang H.X., Ng T.B. (2001). Examination of Lectins, Polysaccharopeptide, Polysaccharide, Alkaloid, Coumarin and Trypsin Inhibitors for Inhibitory Activity Against Human Immunodeficiency Virus Reverse Transcriptase and Glycohydrolases. Planta Med..

[22] Jiratchariyakul W., Wiwat C., Vongsakul M., Somanabandhu A., Leelamanit W., Fujii I., Suwannaroj N., Ebizuka Y. (2001). HIV Inhibitor from Thai Bitter Gourd. Planta Med..

[23] Alves M., Froufe H., Costa A., Santos A., Oliveira L., Osório S., Abreu R., Pintado M., Ferreira I. (2014). Docking Studies in Target Proteins Involved in Antibacterial Action Mechanisms: Extending the Knowledge on Standard Antibiotics to Antimicrobial Mushroom Compounds. Molecules.

[24] Das K., Bauman J.D., Clark A.D., Frenkel Y.V., Lewi P.J., Shatkin A.J., Hughes S.H., Arnold E. (2008). High-resolution structures of HIV-1 reverse transcriptase/TMC278 complexes: Strategic flexibility explains potency against resistance mutations. Proc. Natl. Acad. Sci. USA.

[25] Das K., Clark A.D., Lewi P.J., Heeres J., de Jonge M.R., Koymans L.M.H., Vinkers H.M., Daeyaert F., Ludovici D.W., Kukla M.J. (2004). Roles of Conformational and Positional Adaptability in Structure-Based Design of TMC125-R165335 (Etravirine) and Related Non-nucleoside Reverse Transcriptase Inhibitors That Are Highly Potent and Effective against Wild-Type and Drug-Resistant HIV-1 Varian. J. Med. Chem..

[26] Shivanagoudra S. (2019). Differential Inhibition of Carbolytic Enzymes and Suppression of LPS-Induced Inflammation by Isolated Compounds from Bitter Melon (*Momordica charantia*). Ph.D. Thesis.

[27] Li Z., Xia A., Li S., Yang G., Jin W., Zhang M., Wang S. (2020). The Pharmacological Properties and Therapeutic Use of Bitter Melon (*Momordica charantia* L.). Curr. Pharmacol. Rep..

[28] Bourinbaiar A.S., Lee-Huang S. (1996). The Activity of Plant-Derived Antiretroviral Proteins MAP30 and GAP31 against Herpes Simplex Virus Infectionin Vitro. Biochem. Biophys. Res. Commun..

[29] Lee-Huang S., Huang P.L., Chen H.-C., Huang P.L., Bourinbaiar A., Huang H.I., Kung H. (1995). Anti-HIV and anti-tumor activities of recombinant MAP30 from bitter melon. Gene.

[30] Huang W.-H., Su W.-M., Wang C.-W., Fang Y.-H., Jian Y.-W., Hsu H.-J., Peng C.-W. (2023). *Momordica* anti-HIV protein MAP30 abrogates the Epstein-Barr virus nuclear antigen 1 dependent functions in host cells. Heliyon.

[31] Eryılmaz Pehlivan F. (2021). Bitter Melon: A Multifunctional Medicinal Plant with Powerful Bioactive Compounds. Functional Foods—Phytochemicals and Health Promoting Potential.

[32] Guarniz W.A.S., Canuto K.M., Ribeiro P.R.V., Dodou H.V., Magalhaes K.N., Miranda Sa K., do Nascimento P.G.G., Silva K.L., Passos Sales G.W., Monteiro M.P. (2019). *Momordica charantia* L. Variety from Northeastern Brazil: Analysis of Antimicrobial Activity and Phytochemical Components. Pharmacogn. J..

[33] Braca A., Siciliano T., D’Arrigo M., Germanò M.P. (2008). Chemical composition and antimicrobial activity of *Momordica charantia* seed essential oil. Fitoterapia.

[34] Ahmad R., Aldholmi M., Mostafa A., Alqathama A., Aldarwish A., Abuhassan A., Alateeq L., Bubshait S., Aljaber M., Aldossary S. (2022). A novel green extraction and analysis technique for the comprehensive characterization of mangiferin in different parts of the fresh mango fruit (*Mangifera indica*). LWT.

[35] Ahmad R., Aldholmi M., Alqathama A., Aldossary S., Bubshait S., Aljaber M., Abuhassan A., Aldarwish A., Alateeq L. (2021). Green and novel ultrasonic extraction with UHPLC-MSMS analysis of natural sweetener (Glycyrrhizic acid) from *Glycyrrhiza glabra*; a multifactorial mechanistic evaluation based on statistical analysis. Ultrason. Sonochem..

[36] Ahmad R., Aldholmi M., Alqathama A., Al Nahab H.Z., Almutawah A.I. (2024). A comprehensive LCMS/MS characterization for the green extracted cucurbitane-triterpenoid glycosides from bitter melon (*Momordica charantia*) fruit. Food Chem..

[37] Harnett S.M., Oosthuizen V., van de Venter M. (2005). Anti-HIV activities of organic and aqueous extracts of *Sutherlandia frutescens* and *Lobostemon trigonus*. J. Ethnopharmacol..

[38] Kapewangolo P., Hussein A.A., Meyer D. (2013). Inhibition of HIV-1 enzymes, antioxidant and anti-inflammatory activities of *Plectranthus barbatus*. J. Ethnopharmacol..

[39] Kapewangolo P., Kandawa-Schulz M., Meyer D. (2017). Anti-HIV Activity of *Ocimum labiatum* Extract and Isolated Pheophytin-a. Molecules.

[40] Matotoka M.M., Ndhlala A.R., Masoko P. (2019). In vitro inhibition of HIV-1 reverse transcriptase and anti-inflammatory activities of some herbal concoctions sold in the Limpopo Province. S. Afr. J. Bot..

[41] Barry A.L. (1999). Methods for Determining Bactericidal Activity of Antimicrobial Agents: Approved Guideline.

[42] Kuroda D.G., Bauman J.D., Challa J.R., Patel D., Troxler T., Das K., Arnold E., Hochstrasser R.M. (2013). Snapshot of the equilibrium dynamics of a drug bound to HIV-1 reverse transcriptase. Nat. Chem..

[43] Shelley J.C., Cholleti A., Frye L.L., Greenwood J.R., Timlin M.R., Uchimaya M. (2007). Epik: A software program for pK a prediction and protonation state generation for drug-like molecules. J. Comput. Aided Mol. Des..

[44] Kwofie S., Dankwa B., Odame E., Agamah F., Doe L., Teye J., Agyapong O., Miller W., Mosi L., Wilson M. (2018). In Silico Screening of Isocitrate Lyase for Novel Anti-Buruli Ulcer Natural Products Originating from Africa. Molecules.

