# Supplementary material for: Anti-Infective Activity of Momordica charantia Extract with Molecular Docking of Its Triterpenoid Glycosides
Source: Antibiotics (Basel). 2024 Jun 11;13(6):544. doi: 10.3390/antibiotics13060544 (PMC11200997; doi:10.3390/antibiotics13060544)
Supplement: Supplementary file 1 [file antibiotics-13-00544-s001.zip › antibiotics-3022541-supplementary.pdf]

antibiotics

MDPI

Supplementary data

Table S1. antiviral and antibacterial results for BM fruit parts with extract yields and KVs amounts (*I1; Indian seeds, I2;Indian skin, I3; Indian pith, K1; KSA seeds, K2; KSA skin, K3; KSA pith).

Activity		Sample	I1*	I2	I3	K1	K2	K3
Antiviral	HIV-I RT	Inhibition (%)	72.4	76	75.2	81.6	73.7	79.2
Antibacterial	*Candida albicans* (CA)	MIC (mg/mL)	0.08	0.3	0.08	1	1	0.3
		MBC (mg/mL)	0.3	0.3	0.08	1	1	0.3
	Escherichia coli (EC)	MIC (mg/mL)	0.3	3	0.3	3	3	0.3
		MBC (mg/mL)	1	3	1	3	3	1
	Staphylococcus aureus (SA)	MIC (mg/mL)	3	3	3	3	3	3
		MBC (mg/mL)	3	>3	3	3	3	3
Extract (mg/g)		%yield	2.78	0.09	1.18	5.41	0.18	0.56
KVs amount (µg/L)		KV VIII	2.51	280.95	592.1	0.75	215.32	467.36
		KV X	11.89	229.59	220.39	11.55	1.59	269.55
		KV XI	15.02	223.63	405.32	14.37	94.18	233.76
KVs yield/fruit part (µg/L)			29.42	734.17	1217.81	26.67	311.09	970.67

Table S2. IC50 determination for the active extract based on the antiviral inhibition results.

Extracts (2mg/mL)	Inhibition (%)	IC_{50} (mg/mL)
I1	72.4	
I2	76.0	
I3	75.2	
K1	81.6	0.125
K2	73.7	
K3	79.2	
Negative control (DMSO)	0.0	
Positive control (Doxorubicin)	89.6	0.125

Table S3. bivariate correlation for the extracts Vs DV at P=0.05. *Correlation is significant at the 0.05 level (2-tailed); **Correlation is significant at the 0.01 level (2-tailed).

	DV	I1	I2	I3	K1	K2	K3
DV	1	-0.32	0.670*	0.638*	-0.37	0.33	0.620*
		0.34	0.02	0.03	0.26	0.32	0.04
I1	-0.32	1	0.16	0.05	0.997**	0.23	0.08
	0.34		0.63	0.87	0.00	0.50	0.80
I2	0.670*	0.16	1	0.945**	0.10	0.756**	0.971**
	0.02	0.63		0.00	0.76	0.01	0.00
I3	0.638*	0.05	0.945**	1	0.00	0.898**	0.967**
	0.03	0.87	0.00		0.99	0.00	0.00
K1	-0.37	0.997**	0.10	0.00	1	0.18	0.03
	0.26	0.00	0.76	0.99		0.59	0.94
K2	0.33	0.23	0.756**	0.898**	0.18	1	0.842**
	0.32	0.50	0.01	0.00	0.59		0.00
K3	0.620*	0.08	0.971**	0.967**	0.03	0.842**	1
	0.04	0.80	0.00	0.00	0.94	0.00	

Table S4. Principial component analysis for the DVs with component loadings and, KMO and Bartlett's test at P=0.05.

Components	PC1	PC2
**DV**	0.69	-0.48
**I1**	0.09	0.98
**I2**	0.97	0.06
**I3**	0.99	-0.02
**K1**	0.31	0.99
**K2**	0.87	0.22
**K3**	0.98	0.01
Individual %variance	***58.82***	***31.70***
Cumulative %variance	***58.82***	***90.52***
**KMO and Bartlett's test**		
Kaiser-Meyer-Olkin Measure of Sampling Adequacy		0.48
Bartlett's Test of Sphericity	Approx. Chi-Square	141.33
	df	21
	Sig.	0.00

Table S5. Paired differences for the samples origin Vs individual activity at P=0.05.

**Sample origin Vs individual activity**							
**Pairs**	**Variables**	**Mean**	**SD**	**95% CI**		**t**	**Sig. (2-tailed)**
				**Lower**	**Upper**		
Pair 1	Sample origin - antiviral activity	-72.85	2.96	-75.95	-69.75	-60.32	**0.00**
Pair 2	Sample origin - CA_MIC	3.04	1.69	1.27	4.82	4.40	**0.01**
Pair 3	Sample origin - CA_MBC	3.00	1.75	1.17	4.84	4.21	**0.01**
Pair 4	Sample origin - EC_MIC	1.85	2.27	-0.53	4.23	2.00	0.10
Pair 5	Sample origin - EC_MBC	1.50	2.07	-0.68	3.68	1.77	0.14
Pair 6	Sample origin - SA_MIC	0.53	1.82	-1.38	2.44	0.72	0.51
Pair 7	Sample origin - SA_MBC	0.17	2.32	-2.26	2.60	0.18	0.87
